# Quality, Rigour and Usefulness of Free-Text Comments Collected by a Large Population Based Longitudinal Study - ALSWH

**DOI:** 10.1371/journal.pone.0068832

**Published:** 2013-07-11

**Authors:** Jane Louise Rich, Catherine Chojenta, Deborah Loxton

**Affiliations:** Research Centre for Gender, Health and Ageing, University of Newcastle, NSW, Australia; Research and Development Corporation, United States of America

## Abstract

While it is common practice for health surveys to include an open-ended question asking for additional comments, the responses to these questions are often not analysed or used by researchers as data. The current project employed an automated semantic program to assess the useability and thematic content of the responses to an open-ended free response item included in the Australian Longitudinal Study on Women’s Health (ALSWH) surveys. The study examined the comments of three cohorts of women, born between 1973–78, 1946–51, and 1921–26, from Survey 1 (in 1996) and Survey 5 (in 2007–2009). Findings revealed important differences in the health status of responders compared to non-responders. Across all three cohorts, and at both time points, women who commented tended to have poorer physical health (except for women aged 82–87) and social functioning, experienced more life events, were less likely to be partnered, and (except for women aged 18–23 years) more likely to have higher levels of education, than women who did not comment. Results for mental health were mixed. The analysis revealed differences between cohorts as well as changes over time. The most common themes to emerge for the 1973–78 cohort were *health, time, pregnant* and *work*, for the 1946–51 cohort, the most common themes were *health, life, time* and *work*, while for the 1921–26 cohort, the most common themes were *husband, health* and *family*. The concepts and frequency of concepts changed from the first to the fifth survey. For women in the 1973–78 cohort, *pregnant* emerged as a prevalent theme, while *eating* disappeared. Among women in the 1946–51 cohort, *cancer*, *operation* and *medication* emerged as prevalent themes, while for women in the 1921–26 cohort, the concept *children* disappeared, while *family* emerged. This analysis suggests that free-text comments are a valuable data source, suitable for content, thematic and narrative analysis, particularly when collected over time.

## Introduction

While many health surveys include an open-ended question to allow participants to provide additional comments and information, the responses to these questions are not often examined beyond surface-level analysis. The sole reliance on numerical survey data has been criticised, with researchers pointing to the interpretive value of descriptive comments [1]. The detail provided by free-text comments may offer an important context for participant responses and reveal issues that cannot be identified using purely quantitative surveys [2]. For example, in a study using Likert Scales and free-text comments to assess quality of life among homeless people, students, and a town population, important discrepancies emerged between the two response methods [3]. While homeless people had similar or better quantitative ratings on several of the health-related measures, their free-text accounts revealed a range of unique difficulties, which appeared to contradict the quantitative results, indicating the value of qualitative comments in evaluating health.

Little is known about the health profile of respondents to open-ended questions compared to non-respondents in terms of representativeness of responses. It has been argued that respondents may not be representative of the population surveyed. In a review of free-text comments from a study of maternity care involving recent mothers, and a longitudinal cohort study of UK medical graduates’ careers, Garcia and colleagues [4] found free text respondents were likely to be more articulate, or to have a negative comment to make than non-respondents. These findings suggest that biases may exist in open-ended datasets, however little is known about the more general biases such as demographic factors and overall quality of life.

The potential of free-text responses, particularly in a longitudinal context have increasingly been acknowledged for their utility and narrative potentials. Analysis of free-text data collected over time enables researchers to explore changes in how participants construct meaning across the life course [5], [6], [7], [8], [9]. The growing body of literature that has analysed free-text comments begins to suggest that qualitative data sets are of intrinsic value and can be analysed for more than survey evaluation purposes.

The current project employed an automated semantic program to assess the useability and thematic content of the responses to an open-ended free response item included in the Australian Longitudinal Study on Women’s Health (ALSWH). These data are well suited to interrogation due to the large number of participants from three broadly representative samples [10].

The aims of this project were to assess the quality, rigour and usefulness of the comments collected by the ALSWH in order to validate the targeted analysis of these comments. Additionally the health status of responders compared to non-responders was assessed.

## Methods

This project and the data used in the analysis have received all relevant ethics approvals. Ethics approval was granted by the University of Newcastle (H-076-0795) and the University of Queensland Human Research Ethics Committees (2004000224). Written informed consent was obtained from all participants. This consent procedure was approved by both ethics committees.

The Australian Longitudinal Study on Women’s Health (ALSWH) has been collecting postal survey data from three cohorts of women born between 1973–78 (N = 14247), 1946–51 (N = 13715) and 1921–26 (N = 12804) since 1996. These cohorts have been surveyed by postal survey on approximately a three-yearly basis [11]. In addition to completing quantitative questions on their health and lifestyle, participants are also asked “Have we missed anything?” and given the opportunity to answer the question in an open-ended format. Since the study’s inception in 1996 the ALSWH has been collecting these comments, often using them to provide further insights into quantitative analysis [11] and more recently for in-depth qualitative analysis [8]. This paper presents examples from two separate time points to highlight the validity of free-text comments as data, Surveys 1 and 5 are presented to exemplify this.

Through the use of Leximancer software the researchers were able to provide visual maps of the qualitative data, uncovering the most common themes, words and relationships. This software uses word-association information to elicit emergent concepts from the text [12]. Word frequency and location are used to generate taxonomies which are presented as maps showing the relationships between common concepts. Leximancer software was used to conduct an automatic analysis of the ALSWH qualitative data sets. This analytical tool enables the generation of a taxonomy which is derived from the data itself, offering an efficient way of conceptually mapping, in this case, large data sets. This type of automatic analysis is grounded and exploratory, as the researcher is removed from making any judgements or preconceptions regarding the coding of the data. Leximancer quantifies large text documents using a classification system of learned lexical concepts rather than just keywords [13].

As this project was exploratory in nature, simply to uncover what women wrote about over the study period, all default settings of Leximancer were maintained with one exception. Leximancer by default reads the data in two-sentence blocks, as generally in common language two sentences presented together contain similar content and topics. However, for this project this setting was changed to analyse one-sentence blocks because the nature of free-text comments is that often the sentences are not continuous in content and are rather spontaneous and short in nature.

Descriptive quantitative analyses (t-tests and chi-squares tests) were additionally undertaken to determine demographic and health-related differences between those women who responded to the free-text item and those who did not respond. Physical and mental health and social functioning were measured using the SF-36 Health Survey [14]. The Mental Health Index, Physical Functioning, General Health and Social Functioning subscale scores were compared for those who commented and those who did not comment at Survey 1 and Survey 5 for each cohort. A proportional Life Events score was developed from responses to a life events scale developed from the Norbeck’s [15] life events scale. Demographic measures included partner status (partnered/unpartnered for the 1973–78 Cohort and partnered/unpartnered/widowed for the 1946–51 and 1921–26 Cohorts) and the highest education qualification achieved (Year 12 or less/non-university tertiary/university degree or higher).

## Results

This analysis included data from Survey 1 (in 1996) and Survey 5 (in 2007–2009) of the three ALSWH cohorts. Table 1 shows the number of responses to the open-ended question at each survey. The proportion of women in the 1973–78 Cohort who wrote comments increased over time, while the proportion of women in the 1946–51 Cohort who responded to the free text item remained stable. The 1921–26 Cohort were the most likely to comment and also wrote the most (as measured by number of words).

**Table 1:** Number and percentage of participants who commented by cohort.

Cohort	Number of participants who commented		Total number of words in all ALSWH qualitative datasets	% of cohort to have ever written a free-text comment in the ALSWH
	Survey 1	Survey 5		
1973–78	2423	2415	560 022	46%
1946–51	2447	3731	828 314	49%
1921–26	3399	2481	10897944	56%

### Quantitative Results

#### 1973–78 cohort

At Survey 1 (in 1996), those participants who had significantly lower scores across each of the domains assessed on the SF-36 including mental health, general health, physical functioning and social functioning (Table 2), indicating they had poorer health than those who did not comment were worse off across these domains. Participants who also had significantly higher proportional scores on the life events scale, indicating they experienced more life events than those participants who did not. There were no significant differences across demographic measures for the two groups. The results for Survey 5 (in 2009) for the 1973–78 Cohort can also be found in Table 2. At this time there were no significant differences on the Mental Health Index, however all other subscales of the SF-36 were significantly lower for those women who commented. There was also a significant difference detected for education, with those women who commented more likely to have higher levels of education than those women who did not comment.

**Table 2:** 1973–78 Cohort quantitative measures S1 and S5 for those who commented and those who did not comment.

	Did not Comment	Commented	
Survey 1 (N = 14247)	n = 11826	n = 2425 (17%)	
Mean (SD) SF-36 Scores			
Mental Health Index	68.5 (19.4)	65.5 (18.0)	p<.001
Physical Function	90.8 (14.8)	88.0 (17.3)	p<.001
General Health	69.0 (20.3)	65.5 (21.6)	p<.001
Social Function	77.2 (22.5)	70.3 (25.3)	p<.001
Mean (SD) Proportion of Life events (0–1)	0.16 (0.10)	0.19 (0.11)	p<.001
Education Level			p = .05
Year 12 or less	8217 (70.0%)	1717 (70.6%)	
Non-University tertiary	2090 (17.8%)	386 (15.9%)	
University and higher	1428 (12.2%)	330 (13.6%)	
Partner Status			p = .01
Partnered	2306 (19.7%)	567 (23.2%)	
Un-partnered	9426 (80.3%)	1876 (76.8%)	
**Survey 5 (N = 8254)**	**n = 5771**	**n = 2483 (30.1%)**	
Mean (SD) SF-36 Scores			
Mental Health Index	72.7 (16.3)	71.9 (16.6)	p = .05
Physical Function	91.4 (14.7)	87.5 (18.2)	p<.001
General Health	74.6 (18.1)	72.1 (20.6)	p<.001
Social Function	84.5 (20.5)	78.4 (24.8)	p<.001
Mean (SD) Proportion of Life events (0–1)	0.06 (0.06)	0.06 (0.06)	p<.001
Education Level (Survey 1)			p<.001
Year 12 or less	1123 (19.8%)	369 (15.2%)	
Non-University tertiary	1474 (26.0%)	566 (23.3%)	
University and higher	3075 (54.2%)	1490 (61.4%)	
Partner Status			p = .01
Partnered	4456 (77.4%)	1891 (76.5%)	
Un-partnered	1302 (22.6%)	580 (23.5%)	

#### 1946–51 cohort

At Survey 1 (in 1996), those participants who commented had significantly lower scores across all SF-36 domains, with the exception of social functioning. Those who commented also had significantly higher proportional scores on the life events scale. Women who commented were also more likely to be un-partnered, and had higher levels of education than those women who did not comment. Similar results were reported at Survey 5 for all SF-36 domains, life events scores and demographic measures (see Table 3).

**Table 3:** 1946–51 Cohort quantitative measures S1 and S5 for those who commented and those who did not comment.

	Did not Comment	Commented	
**Survey 1 (N = 13714)**	**n = 11305**	**n = 2409 (17.6%)**	
Mean (SD) SF-36 Scores			
Mental Health Index	72.7 (17.7)	69.9 (19.2)	p<.001
Physical Function	86.0 (17.8)	81.3 (22.2)	p<.001
General Health	72.9 (19.9)	67.3 (23.3)	p<.001
Social Function	74.7 (27.4)	83.0 (22.5)	p<.001
Mean (SD) Proportion of Life events (0–1)	0.11 (0.10)	0.14 (0.10)	p<.001
Education Level			p<.001
Year 12 or less	7471 (66.1%)	1190 (49.4%)	
Non-University tertiary	2104 (18.6%)	566 (23.5%)	
University and higher	1598 (14.1%)	630 (26.2%)	
Partner Status			p<.001
Partnered	9217 (81.5%)	1799 (74.7%)	
Un-partnered	1787 (15.8%)	539 (22.4%)	
Widowed	226 (2.0%)	61 (2.5%)	
**Survey 5 (N = 10532)**	**n = 7736**	**n = 2797 (26.6%)**	
Mean (SD) SF-36 Scores			
Mental Health Index	76.2 (17.2)	74.2 (18.3)	p<.001
Physical Function	81.5 (19.6)	76.6 (23.1)	p<.001
General Health	72.9 (19.8)	67.4 (22.6)	p<.001
Social Function	85.1 (21.7)	77.9 (26.6)	p<.001
Mean (SD) Proportion of Life events (0–1)	0.06 (0.06)	0.08 (0.06)	p<.001
Education Level (Survey 1)			p<.001
Year 12 or less	4930 (64.3%)	1494 (53.9%)	
Non-University tertiary	1524 (19.9%)	629 (22.7%)	
University and higher	1217 (15.9%)	647 (23.4%)	
Partner Status			p<.001
Partnered	6007 (78.2%)	2052 (74.2%)	
Un-partnered	1329 (17.3%)	569 (20.6%)	
Widowed	343 (4.5%)	146 (5.3%)	

#### 1921–26 cohort

At Survey 1 (in 1996), participants who commented had significantly lower scores for Physical Functioning, General Health and Social Functioning scores on the SF-36 compared with women who did not comment. Mental Health Index scores were not significantly different. Significant differences were also detected for higher proportional scores on the life events scale, higher levels of education and partner status. At Survey 5, significant differences were only detected for the Social Functioning score, with women who commented significantly more likely to have lower social functioning scores on the SF-36 compared with women did not write comment. Women who commented were also significantly more likely than women who did not comment to experience a higher proportion of life events, and had a higher level of education (see Table 4).

**Table 4:** 1921–26 Cohort quantitative measures S1 and S5 for those who commented and those who did not comment.

	Did not Comment	Commented	
**Survey 1 (N = 12430)**	**n = 9506**	**n = 2925 (23.5%)**	
Mean (SD) SF-36 Scores			
Mental Health Index	76.6 (17.1)	76.2 (17.7)	p = .279
Physical Function	64.2 (25.6)	60.1 (27.8)	p<.001
General Health	65.8 (21.8)	63.7 (23.2)	p<.001
Social Function	82.5 (24.5)	76.4 (29.1)	p<.001
Mean (SD) Proportion of Life events (0–1)	0.07 (0.08)	0.08 (0.08)	p<.001
Education Level			p<.001
Year 12 or less	7716 (85.5%)	2192 (79.2%)	
Non-University tertiary	987 (10.9%)	395 (14.3%)	
University and higher	318 (3.5%)	182 (6.6%)	
Partner Status			p<.001
Partnered	5225 (56.4%)	1552 (53.2%)	
Un-partnered	811 (8.7%)	355 (12.2%)	
Widowed	3233 (34.9%)	1011 (34.6%)	
**Survey 5 (N = 5601)**	**n = 3080**	**n = 2521 (45%)**	
Mean (SD) SF-36 Scores			
Mental Health Index	77.6 (16.8)	78.7 (16.8)	p = .015
Physical Function	49.0 (28.4)	47.4 (28.2)	p = .037
General Health	61.8 (20.9)	60.5 (21.2)	p = .027
Social Function	75.9 (27.4)	71.3 (29.2)	p<.001
Mean (SD) Proportion of Life events (0–1)	0.10 (0.10)	0.12 (0.10)	p<.001
Education Level (Survey 1)			p<.001
Year 12 or less	2521 (85.1%)	1858 (76.7%)	
Non-University tertiary	327 (11.0%)	396 (16.4%)	
University and higher	114 (3.8%)	168 (6.9%)	
Partner Status			p = .003
Partnered	865 (28.2%)	787 (31.4%)	
Un-partnered	205 (6.7%)	198 (7.9%)	
Widowed	1994 (65.1%)	1524 (60.7%)	

### Qualitative Results

#### 1973–78 leximancer analysis results

**Figure 1:**
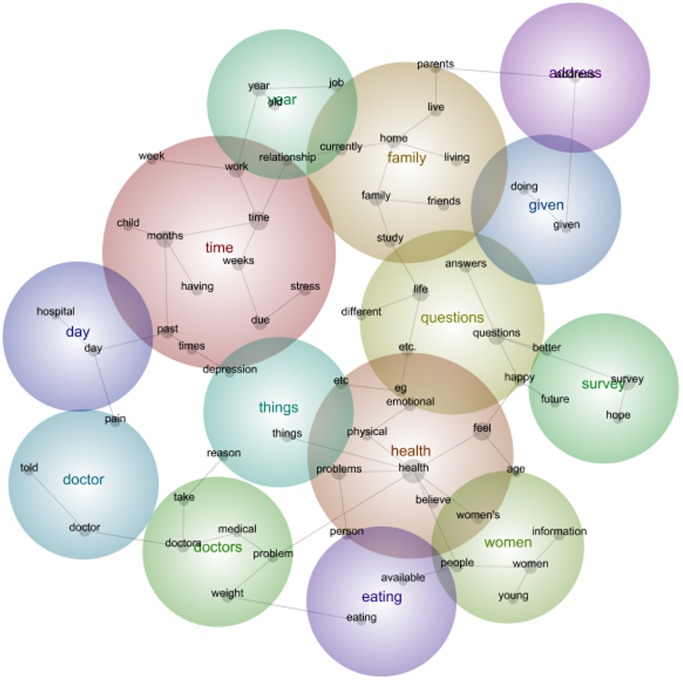
1973–78 cohort qualitative data Leximancer map Survey 1.

The data set of the 1973–78 cohort of the ALSWH includes a diverse range of comments regarding the health and life of the participants. As seen in Figure 1, 1973–78 Cohort Survey 1 data, the Leximancer software maps the themes according to frequency of words and connectedness of words to other words i.e. *pregnancy* and *child*. As a result the maps create concept circles that are heat mapped. The hot (most frequent and most connected i.e. word association) colours through to cooler colours (i.e. the red, orange through to cooler colours such as green and blue to purple) are able indicate meaning and relationships.

In the 1973–78 data set the hottest coloured theme was *Time* followed by *Health* and *Family*. The size of the circles indicates the interconnectedness of each of the themes. For instance, *Time* has overlapped with *Year* (which is not a high frequency colour being Green) however the size highlights the importance of the relationship between *Time* and *Year*. So it is clear that through a visual analysis of the Leximancer map the warmth of the colour is important but also the interconnectedness between the circles is important when exploring the comments made by the women.

Within the main theme of *Time*, key words such as *time, months, stress, work, due, past, times, weeks, child, week, depression, relationship* derived from the data. The results of this map in comparison to the Survey 5 map highlight changes over time expressed by the participants.

**Figure 2:**
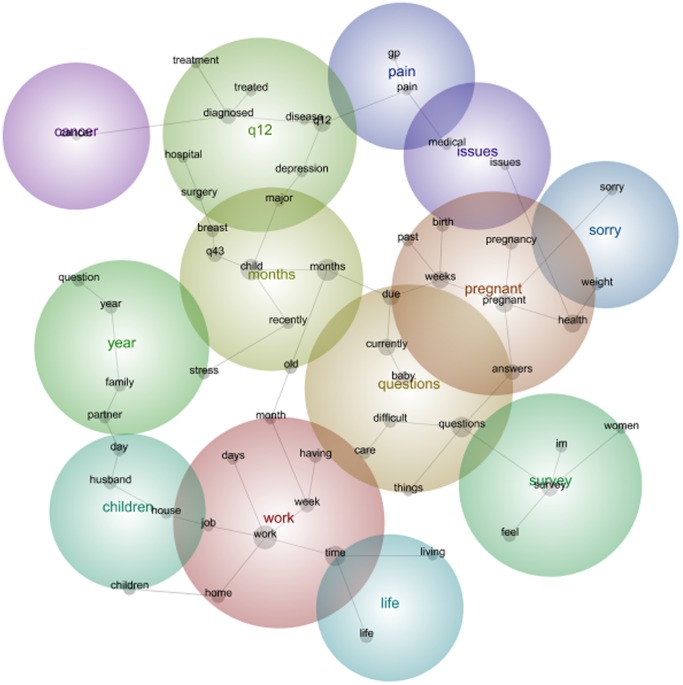
1973–78 cohort qualitative data Leximancer map Survey 5.

When the 1973–78 Cohort was surveyed approximately 15 years later, the themes and concepts showed transitions and developments. In this map, Figure 2, the main theme is *Work*, followed by *Pregnant, Questions, Months* and *Survey*. This indicates that the women used the free-text space to comment on the survey and the questions in the study. However, this also indicates that issues surrounding *Work* and *Pregnancy* are dominating these women’s comments, which is dissimilar to the earlier survey where *Pregnancy* was not a theme.

Within the theme of *Work*, significant concepts words included *work, time, week, home, having, days, months, job.*

#### 1946–51 leximancer analysis results

**Figure 3:**
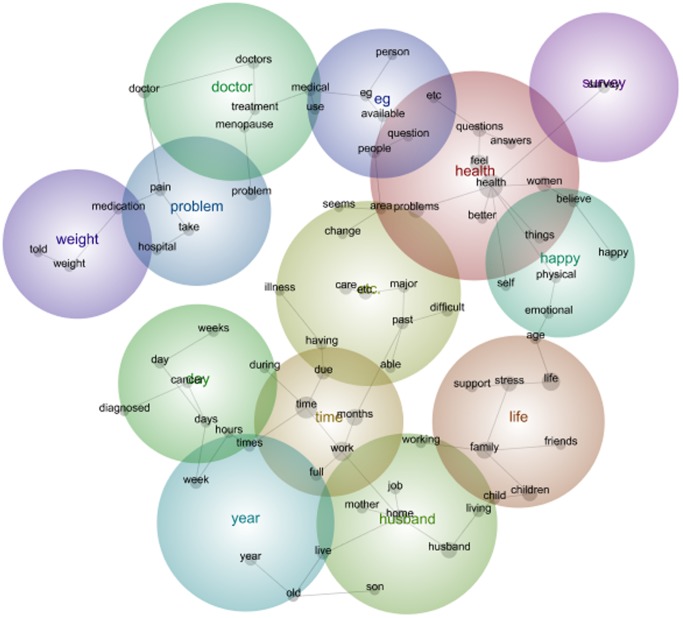
1946–51 cohort qualitative data Leximancer map Survey 1.

The map of the 1946–51 Cohort data are a rich collection of assorted comments, raising some similar and different issues to the younger cohort. In the first survey of this cohort, the most common theme was Health, followed by Life and Time. Within the theme of *Health,* concept words found within that theme include *health, feel, women, people, better, answers, things, believe, question* and *etc*. (Figure 3). These concepts indicate that the 1946–51 Cohort communicate with the ALSWH researchers about their feelings and health related to the survey items.

**Figure 4:**
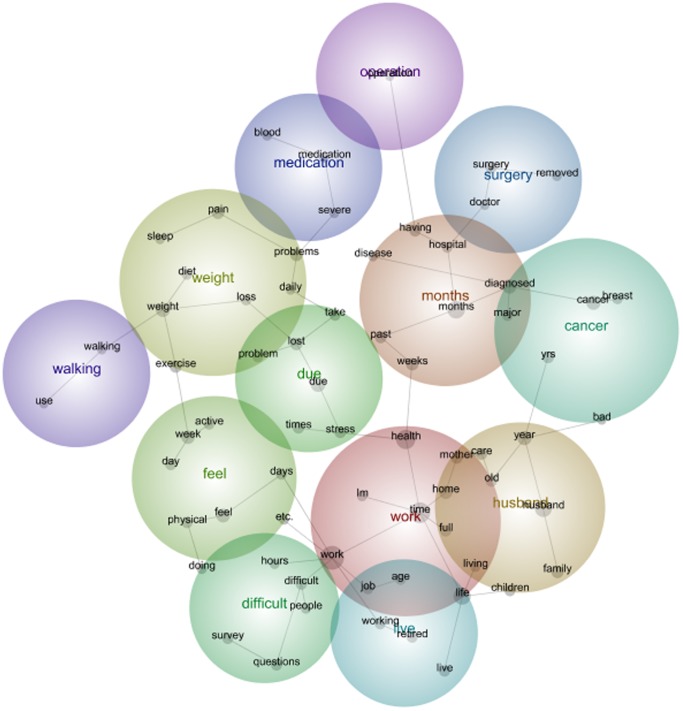
1946–51 cohort qualitative data Leximancer map Survey 5.

It is interesting to note that in survey 5 the main themes, or the hottest colours, have changed slightly. Over time, *Work* has shifted to become the most prominent theme derived from the data. *Work* is interlinked with themes such as *Husband, Live* and *Difficult.* Within the theme of *Work* are the concepts, *time,health, life, home, mother, full, living, age and job* (Figure 4). This interconnection emphasises the challenges of home life and work life for women in mid-life.

Each of the above examples illustrates how life events, health and emotions were often written about together by the 1946–51 Cohort participants.

#### 1921–26 leximancer analysis results

The map of the 1921–26 Cohort comments is the largest collection of ALSWH comments. As with the other datasets, the 1921–26 Cohort data are rich in diversity of themes, contents and experiences. The participants in this cohort generally wrote longer comments (and often in letter style) and are were likely to write more frequently, that is,. several survey waves.

**Figure 5:**
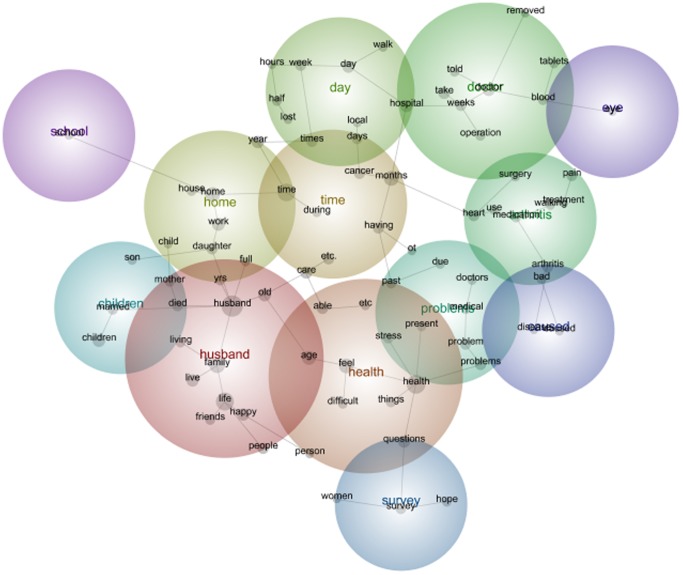
1921–26 cohort qualitative Leximancer map Survey 1.

**Figure 6:**
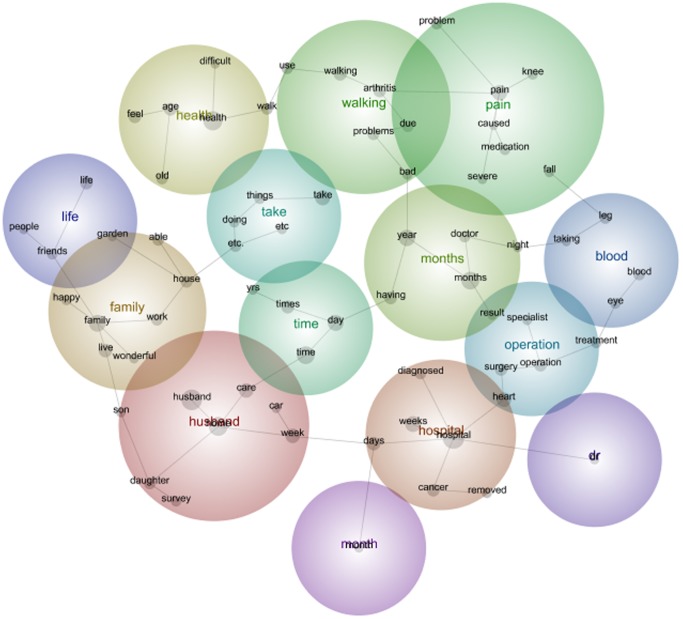
1921–26 cohort qualitative data Leximancer map Survey 5.

In the 1921–26 first survey the theme of *Husband* was the warmest colour. Included in this theme were the concepts *life, family, happy, old, live, friends, living, died, mother, people, age, years and full* (Figure 5). It is interesting, that at Survey 5, twelve years after the first survey, the theme *Husband* remained the hottest theme (Figure 6).

In 2008, under the theme of *Husband,* the concepts changed since 1996. These newer concepts include *home, week, care, daughter, son,* and *car*. There is a noticeable shift in the types of comments these older women were writing in 2008 in comparison to the 1996 data. For example, the term ‘children’ has disappeared from this data set in 2008 and been replaced by the emergent term ‘family’ as these women moved from their mid-seventies into very old age.

## Discussion

Across all cohorts, women who wrote free text comments tended to have poorer physical health and to have higher levels of education. Results for psychological wellbeing were inconsistent. In addition those results for the 1921–26 Cohort at Survey 5 were inconsistent, possibly due to the number of women who have died or withdrawn from the study by Survey 5, due to the high numbers of participants who commented in this cohort by this time.

Examination of the concept maps revealed differences between the three cohorts. The most common themes were for the 1973–78 Cohort, were *health, time, pregnant* and *work,* for the 1946–51 Cohort, were *health, life, time* and *work*, and for the 1921–26 Cohort, were *husband, health* and *family*. All cohorts wrote about health related concepts, as would be expected in a health survey. Common themes included: eating and pain from the 1973–78 Cohort, weight, cancer and medication from the 1946–51 Cohort, and arthritis, eye and pain from the 1921–26 Cohort.

The concepts and frequency of concepts changed from the first to the fifth survey. For women in the 1973–78 Cohort this included the emergence of *pregnant* and the disappearance of *eating* as prevalent themes among women moving from their early twenties to their early thirties. For women in the 1946–51 Cohort this included the emergence of *cancer*, *operation* and *medication* as prevalent themes among women moving from their middle forties to their fifties. And for the 1921–26 Cohort, this included the disappearance of the concept *children* and emergence of the concept *family* among women moving from their mid seventies into very old age. The maps (see Figures 1, 2, 3, 4, 5, 6) also illustrate the intersections between concepts and the changes that occur in these intersections over time.

### Assessing Quality through the Kitto et al (2008) Framework

In assessing this data the authors have applied a framework explained by Kitto and colleagues [16] which examines qualitative validity. This framework sets some ground rules for assessing quality in qualitative data and was recently published in an edition of the Medical Journal of Australia, applied below [16].

#### Clarification

This aim of this paper was to reveal via a Leximancer analysis what the women in the ALSWH write about at the end of a survey, the health differences between those who write and those who do not write, and to assess whether or not the data collected is a viable option for research.

#### Justification

This was an important research question to ask of the ALSWH data as it has never been asked before. Surveys and questionnaires often ask their participants if there is anything else they would like to tell the research team, but, to date, this is a largely untapped source of research data. Often, these data are only used only for quality assurance or evaluation of survey items.

#### Procedural rigour

All processes of the data collection have been documented and approved by relevant ethical boards. The data used in this analysis have not been edited or changed by researchers, apart from being typed into a database. Leximancer is an automated program based on word count and word association; therefore, this analysis is relatively unbiased and naturally derived from the original data.

#### Representativeness

This study is a nation-wide longitudinal Australian study. The sample is broadly representative of the population of Australian women in included age groups. Not every participant has written comments; however, many have, as Table 1 details.

#### Interpretation

This data analysis was conducted by an automated computer program, Leximancer, which consequently has reduced researcher interpretation bias.

#### Reflexivity and evaluative rigour

This analysis strongly confirmed the views of the researchers, that in fact, qualitative free text comments are a valid source of data, particularly when collected over time. These type of data can be used for content, thematic, narrative and other forms of qualitative analysis and are especially useful when collected over time.

#### Transferability

The authors conclude that this method can be transferred to similar contexts. As mentioned, the process of collecting free-text comments is common among population and epidemiological studies; this study concludes that these qualitative data can be an asset to these studies and further understandings of particular phenomena.

### Contributions and Limitations of this Study

This study was unique in its protocol and analysis. Never before have free text comments collected over a 15 year time period been subjected to a Leximancer analysis. This study has the capacity to encourage other survey based studies to analyse qualitative comments. An important limitation of this study is that only comments written by women who participate in the ALSWH have been analysed, therefore there may be other themes that have not been included in this study, which are of importance to women in Australia who did not write at the back of the ALSWH surveys. A further limitation has been identified by the quantitative analysis. Broadly speaking, those participants who commented were of poorer health, un-partnered and had higher levels of education compared with women who did not comment. Nonetheless, these comments provide valuable insight in to the health, wellbeing and lifestyle of Australian women over time.

### Conclusion

This analysis of free text comment from a longitudinal study is novel, and as far as the researchers have found, it has never before been validated that free-text comments collected over time can be used as an effective and justified data source. Free text response offers a rich source of data suitable for content, thematic and narrative analysis, particularly when collected over time.
